# The Spectrum of Pathogenic Yeast Infection in a Tertiary Care Hospital in Assam, India

**DOI:** 10.7759/cureus.31512

**Published:** 2022-11-14

**Authors:** Debarati Saha, Ajanta Sharma, Nilakshi Borah, Dibyajyoti Saikia

**Affiliations:** 1 Microbiology, All India Institute of Medical Sciences, Guwahati, IND; 2 Microbiology, Gauhati Medical College and Hospital, Guwahati, IND; 3 Microbiology, State Cancer Institute, Government Medical College and Hospital, Guwahati, IND; 4 Pharmacology, All India Institute of Medical Sciences, Guwahati, IND

**Keywords:** bloodstream infection, emerging yeast, cryptococcus, candida, antifungal susceptibility

## Abstract

Context: In view of the growing incidence of pathogenic yeast infection all over the world, this study was undertaken to understand its etiology and epidemiology in Assam.

Aims: To characterize and study the antifungal susceptibility pattern of the pathogenic yeasts from the clinical samples.

Settings and Design: The study was a hospital-based cross-sectional study.

Methods and Material: 150 patients were enrolled in the study and from which clinical samples were collected. A total of 83 samples showing the growth of yeast in culture were included in the study. The yeasts were identified by conventional and BioMerieux ID 32C and VITEK 2^TM^. Antifungal susceptibility test was done by disk diffusion method as per Clinical and Laboratory Standards Institute (CLSI), M44-A2.

Statistical analysis used: Data was analyzed using statistical software Epi-Info 7.1.2.0 (2013; CDC, Atlanta, USA). For comparison of categorical data, the Chi-square test or Fisher exact test was used. A value of *p* less than 0.05 was considered statistically significant.

Results: The most affected population was the age group of ≤10 years (32.5%) with male preponderance (67.5%). Yeasts were mostly isolated bloodstream infections (49.3%). The major risk factor was prolonged antibiotic intake. Predominant yeast isolates were *Candida albicans* (43.4%) followed by* Candida tropicalis *(19.3%). Emerging yeasts like *Kodaemea ohmeri* (4.8%), *Pichia anomala* (2.4%), and *Candida auris* (1.2%) were also isolated. Amphotericin B was effective against all yeast isolates. All the isolates of *Candida krusei *were resistant to all the azoles.

Conclusions: The study reflects that there is a growing incidence of emerging yeast infections and efforts are to be made for their identification and antifungal susceptibility testing for the initiation of appropriate therapy.

## Introduction

Infections have dramatically increased over the last few decades due to pathogenic yeasts. The increasing risk of opportunistic fungal infections is due to growing numbers of immunocompromised hosts, including AIDS patients, the abuse of broad-spectrum antibiotics, immunosuppressive agents after organ transplantation, and cancer chemotherapy [1,2]. Apart from *Candida *species, various fungi like *Trichosporon, Cryptococcus neoformans*, and some emerging yeasts have been widely reported [3,4]. These opportunistic yeasts are ubiquitous and they can be acquired from the normal endogenous flora (*Candida*) or obtained from host surroundings (*Cryptococcus*) [5]. The ARTEMIS global program is one of the most comprehensive and long-running fungal surveillance programs. This program generates massive amounts of data that have been externally validated and that can be used to identify temporal and geographic trends in the species distribution of pathogenic yeasts [6]. On the global scale, *Candida* species accounted for 95-97% of all clinical isolates in each study year from 1997 to 2005 [6]. *Candida albicans *was the most frequent (overall, 65.6% of all Candida species), followed by *Candida glabrata* (11.2%), *Candida tropicalis *(7.0%), and *Candida parapsilosis *(5.8%). Among the non-candidial yeast, *C. neoformans* (31.2%) was most frequent, followed by *Saccharomyces *species (9.6%), *Trichosporon *species (6.7%), and *Pichia anomala *(2.5%) [7]. In India also prevalence rate of* Candida* infection was reported to be 6% in a 5-year study (2001-2005) [8].There is an increasing drift of disease by non-albicans *Candida *(NAC) [8,9]. *C. neoformans *is a saprophytic encapsulated haploid yeast that is distributed worldwide in association with avian excreta, mostly pigeons [10]. There is a rising trend of cryptococcosis in India which is posing a serious threat. As per different Indian studies, *Cryptococcal* infection was seen in the range of 2.09-53.1% [10-12]. Fluconazole resistance amongNAC ranges from 14.5% to 41.6% [10,11]. *Candida auris* has caused increased clinical attention due to its multidrug resistance [13-15]. To manage patients suffering from yeast infections, antifungal susceptibility has become important for initiating treatment. Antifungal susceptibility testing using a disk diffusion method is a simple, rapid, and cost-effective method for screening the susceptibility pattern of yeast [16]. Although the epidemiology of pathogenic yeast infection has been reported from various parts of India, there is a paucity of data on pathogenic yeast infection in Assam [17]. Also, there is a lack of data concerning the in-vitro susceptibility of pathogenic yeasts and yeast-like fungi from Assam. Considering the lack of availability of local epidemiological data, this study was undertaken to characterize and study the antifungal susceptibility pattern of the pathogenic yeasts from the clinical samples.

## Materials and methods

The study was conducted in the Department of Microbiology, Gauhati Medical College and Hospital, Guwahati, for a period of one year from June 2017 to May 2018. It was a cross-sectional study. One hundred and fifty patients were enrolled in the study and from whom clinical samples were collected. The samples were collected from the patients attending OPD and indoors during the study period. Eighty-three samples showing growth of yeast in culture were further processed for their identification and antifungal susceptibility testing. Approval from the Institutional Ethical Committee (IEC) was obtained before the commencement of the study. The clinical specimens (blood, CSF, other body fluids, and sputum) were collected under aseptic conditions. The samples were processed as per standard protocol [18]. Direct microscopy (Gram Stain, Indian Ink) was done. Isolation was done from the culture in Sabouraud’s dextrose agar with chloramphenicol (0.05 mg/ml). Identification of different *Candida *species was done by Germ tube test, Corn Meal Agar morphology (Dalmau Technique), HiChrom Candida Agar, sugar assimilation and fermentation tests and using Biomerieux ID 32C, and VITEK 2^TM ^with YST card for yeast identification system. For confirmation of *Cryptococcus *species, urease hydrolysis tests and phenol oxidase tests were done. Antifungal disk diffusion susceptibility test was done as per the Clinical and Laboratory Standards Institute (CLSI) protocol (M44-A2, CLSI, USA) to find the susceptibility pattern of the fungal growths (Table 1). Institutional Review Board- Institutional Ethics Committee of Gauhati Medical College and Hospital and approval number is 190/2007/Pt-1/EC/120.

Interpretative criteria [19]

**Table 1:** Interpretive criteria-ELLIS-2015

Antifungal agents	Amphotericin B	Fluconazole	Itraconazole	Voriconazole
Susceptible(S)	≥ 15 mm	≥ 19 mm	≥ 23 mm	≥ 17 mm
Susceptible-dose dependent (S-DD)	10-14 mm	15-18 mm	14-22 mm	14-16 mm
Resistant (R)	< 10 mm	≤ 14 mm	≤ 13 mm	≤ 13 mm

## Results

Eighty-three samples showing the growth of yeast in culture were further processed for their identification and antifungal susceptibility testing. Maximum number of the patients (32.5%) were of ≤10 years of age followed by 24.1% in the >60 years age group. This shows that higher incidence of infection with pathogenic yeast was common in the extremes of age. Majority (49.3%) of the patients presented with bloodstream infection (BSI). The second common clinical presentation was bronchopneumonia and pleural effusion (36.1%) followed by meningitis (12%) and tuberculosis (2.4%).

With regard to the risk factors, 88% of the patients in this study had a history of prolonged antibiotic intake (≥7 days) within the last 1 month and it was the leading risk factor in this study. The antibiotics that were used were cephalosporins, carbapenems, and aminoglycosides mostly. Another leading risk factor in this study was ≥7 days stay in the hospital (88%). The second commonest risk factor was indwelling devices like central line, intravenous cannula, and urinary catheter. 92.8% of the patients had history of indwelling devices.

In this study, the maximum number of isolates (Table 2) was *C. albicans* (36; 43.4%). Among the NAC, *C. tropicalis* (19.3%) was the most common isolate.* C. neoformans* was the third commonest yeast isolated from all the CSF samples.* Kodaemia ohmeri* was found in 4.8% of cases and all the isolates were from BSI in neonates. In the present study, the third most common *Candida* species was *C. parapsilosis* (10.8%). In this study, 2.4% of the cases yielded *P. anomala* and all were isolated from blood in neonates.

**Table 2:** Frequency of isolation of different yeasts

Isolates	Frequency (%)	Percentage (%)
Candida guilliermondii	1	1.2
Candida auris	1	1.2
Candida parapsilosis	9	10.8
Candida albicans	36	43.4
Candida famata	2	2.4
Candida krusei	3	3.6
Cryptococcus neoformans	9	10.8
Candida tropicalis	16	19.3
Kodamaea ohmeri	4	4.8
Pichia anomala	2	2.4
Total	83	100

The antifungal susceptibility pattern showed that all the yeast isolates (100%) were susceptible to amphotericin B. All the isolates of *C. krusei *and *Candida auris* were resistant to all the azoles. 91.7% and 83.3% of the *C. albicans* were sensitive to fluconazole and voriconazole, respectively. In this study, 56.3%, 81.3%, and 68.8% of *C. tropicalis* were sensitive to fluconazole, voriconazole, and itraconazole, respectively (Table 3).

**Table 3:** Sensitivity of isolates to antifungal agents

Isolates	Fluconazole (%)	Voriconazole (%)	Itraconazole (%)	Amphotericin B (%)
Candida guilliermondii	0	100	0	100
Candida auris	0	0	0	100
Candida parapsilosis	77.8	77.8	88.9	100
Candida albicans	91.7	83.3	88.9	100
Candida famata	0	100	0	100
Candida krusei	0	0	0	100
Cryptococcus neoformans	88.9	88.9	100	100
Candida tropicalis	56.3	81.3	68.8	100
Kodaemia ohmeri	50	100	50	100
Pichia anomala	50	50	50	100

## Discussion

This study was done to determine the occurrence of pathogenic yeast infections and their antifungal susceptibility pattern in Assam. Most of the studies showed the growing incidence of pathogenic yeasts in immunocompromised patients. The present study showed a higher incidence of infection with pathogenic yeast was common in the pediatric age group as well as in the geriatric age group as the immunity is low in the extremes of age, which is in concordance with the study by Kim et al. (2016) who reported that 75.8% of the patients were in the age group of >60 years of age [20]. The present study showed a higher incidence in males. However, the finding was not statistically significant. A similar finding was also reported by Shaik et al. (2016) who also found that 62.6% males and 37.3% females had an infection due to pathogenic yeasts [13]. In the present study, the majority (49.3%) of the patients presented with BSI. The second most common clinical presentation was bronchopneumonia and pleural effusion (36.1%) followed by meningitis (12%) and tuberculosis (2.4%). This data was statistically significant. Shaik et al (2016) found medical causes 21 (14%) which included pleural effusion, bronchopneumonia, and tuberculosis associated with the isolation of species [13]. With regard to the risk factors, as consistent with the study by Juyal et al. (2013) found that 61.36% of the patients had a history of prolonged broad-spectrum antibiotic use [21]. The second commonest risk factor was indwelling devices like central line, intravenous cannula, and urinary catheter. 92.8% of the patients had a history of indwelling devices. Juyal et al. (2013) in their study found that 64.39% of the patients had a history of indwelling catheters [21]. The study of Kim et al. (2016) reported a higher incidence of fungal isolates in blood after urine, which was consistent with our study [20]. A study by Shaik et al (2016) found 26.9% of fungal isolates in respiratory samples [13].

In the present study maximum preponderance of isolates was *C. albicans* and this finding is in concordance with Kim et al., 2016 (48.6%) [20]. Among the NAC, *C. tropicalis* was the most common isolate. Chakrabarti et al. (2015) also found* C. tropicalis* as the most common isolate with an isolation rate of 31.2% and 41.6% [22].

The study of Taj-Aldeen et al. (2006) reported *K. ohmeri* in neonatal candidemia [23]. In the present study, the third most common *Candida* species was* C. parapsilosis* (10.8%). This finding corresponds with the finding of Chakrabarti et al. (2015) who also reported *C. parapsilosis* to be the third most common *Candida* species (10.9%) [22].

The study showed that *P. anomala* isolates were isolated from blood in neonates. This shows a higher incidence of infection in neonates which also corresponds with the findings of the study by Pasqualotto et al. (2005) [24]. Shaik et al. found a similar isolation rate of *C. krusei* (4.7%) [13].

The antifungal susceptibility pattern showed that all the yeast isolates were sensitive to amphotericin B, which correlates with the report of Shaik et al (2016) [13]. Shaik et al. (2016) also reported that 76.6% and 63.3% of *C. albicans* were sensitive to fluconazole and voriconazole, respectively, which is in concordance with the present study [13]. As fluconazole has been found to be widely resistant so discovery of benzylthio-analogs of fluconazole as a potent antifungal [25].

In the present study, it was not possible to identify *K. ohmeri, C. auris, P. anomala* by conventional methods, and they were identified by automated culture methods only. Therefore, conventional and automated methods are required for the identification of pathogenic yeast. Variation of species distribution is also dependent on variation in methods adopted in different studies.

The limitations of the study were that the characterization and antimicrobial susceptibility of the organisms are done using conventional methods and automated blood culture system but molecular characterization could not be done. The sample size is also less as the study was done for only a year.

## Conclusions

To conclude, the incidence of pathogenic yeast infections is increasing with a gradual increase in infection with some emerging yeasts, many of them showing resistance to commonly used antifungals, making it necessary for their speciation. Hence, identification of the yeasts up to their species level is of utmost importance for the commencement of appropriate antifungal therapy.

## References

[1] Miceli MH, Diaz JA, Lee SA (2011). Emerging opportunistic yeast infections. Lancet Infect Dis.

[2] Sadeghi-Ghadi Z, Vaezi A, Ahangarkani F, Ilkit M, Ebrahimnejad P, Badali H (2020). Potent in vitro activity of curcumin and quercetin co-encapsulated in nanovesicles without hyaluronan against Aspergillus and Candida isolates. J Mycol Med.

[3] Denning DW, Stevens DA, Hamilton JR (1990). Comparison of Guizotia abyssinica seed extract (birdseed) agar with conventional media for selective identification of Cryptococcus neoformans in patients with acquired immunodeficiency syndrome. J Clin Microbiol.

[4] Wolf DG, Falk R, Hacham M (2001). Multidrug-resistant Trichosporon asahii infection of nongranulocytopenic patients in three intensive care units. J Clin Microbiol.

[5] Perfect JR, Casadevall A (2006). Fungal molecular pathogenesis: what can it do and why do we need it?. Heitman J, Filler SG, Edwards JE Jr, Mitchell AP.

[6] Pfaller MA, Diekema DJ, Gibbs DL (2007). Results from the ARTEMIS DISK Global Antifungal Surveillance Study, 1997 to 2005: an 8.5-year analysis of susceptibilities of Candida species and other yeast species to fluconazole and voriconazole determined by CLSI standardized disk diffusion testing. J Clin Microbiol.

[7] Lee MK, Yong D, Kim M, Kim MN, Lee K (2010). Species distribution and antifungal susceptibilities of yeast clinical isolates from three hospitals in Korea, 2001 to 2007. Korean J Lab Med.

[8] Xess I, Jain N, Hasan F, Mandal P, Banerjee U (2007). Epidemiology of candidemia in a tertiary care centre of north India: 5-year study. Infection.

[9] Fakhim H, Vaezi A, Javidnia J (2020). Candida africana vulvovaginitis: Prevalence and geographical distribution. J Mycol Med.

[10] Chander J (2018). Cryptococcosis. Textbook of Medical Mycology. 4th Edition.

[11] Lakshmi V, Sudha T, Teja VD, Umabala P (2007). Prevalence of central nervous system cryptococcosis in human immunodeficiency virus reactive hospitalized patients. Indian J Med Microbiol.

[12] Satpute MG, Telang NV, Litake GM, Niphadkar KB, Joshi SG (2006). Prevalence of cryptococcal meningitis at a tertiary care centre in Western India (1996-2005). J Med Microbiol.

[13] Shaik N, Penmetcha U, Myneni RB, Yarlagadda P, Singamsetty S (2016). A study of identification and antifungal susceptibility pattern of Candida
species isolated from various clinical specimens in a tertiary care teaching
hospital, Chinakakani, Guntur, Andhra Pradesh, South India. Int J Curr Microbiol App Sco.

[14] Arastenfer A, Fang W, Badali H (2018). Low cost tetraplex PCR for the global spreading multi-drug resistant fungus, Candida auris and its phylogenetic relatives. Front Microbiol.

[15] Chatterjee S, Alampalli SV, Nageshan RK, Chettiar ST, Joshi S, Tatu US (2015). Draft genome of a commonly misdiagnosed multidrug resistant pathogen Candida auris. BMC Genomics.

[16] Jeon S, Shin JH, Lim HJ (2021). Disk diffusion susceptibility testing for the rapid detection of fluconazole resistance in Candida isolates. Ann Lab Med.

[17] Das P, Lahari S, Nath R, Phukan SK (2016). Species distribution & antifungal susceptibility pattern of oropharyngeal Candida isolates from human immunodeficiency virus infected individuals. Indian J Med Res.

[18] Collee JG (1996). Specimen collection culture containers and media. Mackie & McCartney Practical Medical Microbiology, 14th Edition.

[19] Meis J, Petrou M, Bille J, Ellis D, Gibbs D, the Global Antifungal Surveillance Group (2000). A global evaluation of the susceptibility of Candida species to fluconazole by disk diffusion. Diagn Microbiol Infect Dis.

[20] Kim GY, Jeon JS, Kim JK (2016). Isolation frequency characteristics of Candida species from clinical specimens. Mycobiology.

[21] Juyal D, Sharma M, Pal S, Rathaur VK, Sharma N (2013). Emergence of non-albicans Candida species in neonatal candidemia. N Am J Med Sci.

[22] Chakrabarti A, Sood P, Rudramurthy SM (2015). Incidence, characteristics and outcome of ICU-acquired candidemia in India. Intensive Care Med.

[23] Taj-Aldeen SJ, Doiphode SH, Han XY (2006). Kodamaea (Pichia) ohmeri fungaemia in a premature neonate. J Med Microbiol.

[24] Pasqualotto AC, Sukiennik TC, Severo LC, de Amorim CS, Colombo AL (2005). An outbreak of Pichia anomala fungemia in a Brazilian pediatric intensive care unit. Infect Control Hosp Epidemiol.

[25] Motahari K, Badali H, Hashemi M (2018). Discovery of benzylthio analogs of fluconazole as potent antifungal agents. Future Med Chem.

